# Integrating Energy Efficiency into Healthcare Operations: A Discrete-Event Simulation Approach for Surgical Pathways

**DOI:** 10.3390/healthcare14091134

**Published:** 2026-04-23

**Authors:** Francesco Sferrazzo, Beatrice Marchi, Anna Savio, Andrea Roletto, Simone Zanoni

**Affiliations:** 1 Department of Mechanical and Industrial Engineering, Università degli Studi di Brescia, 25123 Brescia, Italy; f.sferrazzo@studenti.unibs.it (F.S.); beatrice.marchi@unibs.it (B.M.); anna.savio@unibs.it (A.S.); andrea.roletto@unibs.it (A.R.); 2 Department of Civil, Environmental, Architectural Engineering and Mathematics, Università degli Studi di Brescia, 25123 Brescia, Italy

**Keywords:** healthcare, energy efficiency, discrete-event simulation, operating rooms, sustainability, hospital management, FlexSim Healthcare

## Abstract

**Background/Objectives:** Healthcare facilities are among the most energy-intensive public buildings, yet hospital decision-support models rarely integrate energy-related performance indicators alongside operational metrics. This study aims to address this gap by developing a discrete-event simulation framework capable of jointly evaluating clinical efficiency and energy consumption in elective orthopedic surgical pathways. **Methods:** A comprehensive discrete-event simulation model was developed to represent the diagnostic imaging and orthopedic surgical process. The model was parameterized using a hybrid data-collection approach that combined clinical activity data, scientific literature, and expert judgment. Energy consumption was modeled by differentiating fixed loads, such as heating, ventilation, and air-conditioning systems and lighting, from activity-dependent loads associated with diagnostic and surgical equipment. Baseline performance was assessed and compared with alternative scenarios for organizational and technological improvements. **Results:** The analysis showed that fixed infrastructural loads, particularly HVAC systems, were the main drivers of per-patient energy consumption, with inefficient space utilization and prolonged idle times. Scenario analysis demonstrated that organizational interventions, such as increasing operating room throughput and optimizing MRI scheduling, can substantially reduce energy intensity by diluting fixed loads and decreasing idle consumption. Technological interventions, such as replacing conventional surgical lamps with LED systems, produced smaller but still beneficial reductions. The combined implementation of organizational and technological strategies yielded the greatest overall improvement. **Conclusions:** Integrating energy metrics into discrete-event simulation provides effective support for hospital decision-making by revealing the interaction between workflow design, resource utilization, and environmental performance. The findings indicate that organizational redesign, particularly when combined with technological upgrades, can significantly improve both operational efficiency and sustainability in hospital settings. This study highlights discrete-event simulation as a promising tool for energy-aware healthcare planning.

## 1. Introduction

Environmental sustainability has established itself as a priority in global policy debates, driven by the escalating demand for energy and the persistent reliance on fossil fuel-based systems, which remain the primary drivers of climate change [1]. While international decarbonization efforts are underway, the transition comes with significant economic implications; achieving a net-zero economy requires a fundamental transformation of the global energy mix to mitigate costs and unlock potential growth opportunities [2].

Within this broader framework, the healthcare industry exhibits a structural paradox: while essential for societal well-being, the sector significantly contributes to environmental degradation [3]. Recent global assessments indicate that healthcare is responsible for approximately 4.4% of total net emissions, a footprint comparable to that of the fifth-largest emitting country in the world [4]. Furthermore, pollution resulting from healthcare services inadvertently exacerbates public health challenges, creating a feedback loop that increases healthcare demand [5].

Energy consumption in healthcare facilities is characterized by high intensity and poor integration into operational decision-making [6]. This issue is particularly acute in the Operating Room (OR). Although ORs typically occupy a small fraction of a hospital’s total footprint, they are responsible for a disproportionately high share of energy consumption-often three to six times more than other hospital wards [7]. This intensity is largely driven by stringent HVAC (Heating, Ventilation, and Air Conditioning) requirements for infection control, which necessitate continuous operation even during non-productive hours [8]. Consequently, operating theatres represent a critical area for implementing energy reduction strategies without compromising patient safety [9].

To address these complexities, Discrete-Event Simulation (DES) has been widely acknowledged as a robust scientific approach for analyzing healthcare systems with stochastic behavior and resource constraints [10]. DES allows managers to evaluate scenarios and optimize patient flows without disrupting real-world operations [11]. However, a systematic review of the literature reveals a significant methodological gap: existing simulation studies focus predominantly on operational KPIs, such as waiting times and throughput, with limited explicit integration of dynamic energy consumption metrics [12]. This lack of integration restricts the ability of decision-makers to evaluate trade-offs between operational efficiency and environmental performance [13].

Building on these findings, this project aims to develop an integrated discrete-event simulation model within FlexSim Healthcare. The research focuses on orthopedic surgery, a discipline identified as having one of the highest carbon footprints due to the intensive use of power tools, consumables, and sterilization processes [14]. The proposed model maps the complete surgical pathway, assigning energy profiles to medical equipment and infrastructural systems based on verified clinical duty cycles.

The core novelty of this study lies in a paradigm shift: repurposing Discrete-Event Simulation (DES), traditionally a tool for operational throughput, into a dedicated engine for environmental sustainability. While previous research focuses on reducing waiting times or increasing patient flow, this framework treats energy consumption not as a static building overhead, but as a dynamic clinical variable. By mapping specific equipment duty cycles directly onto the stochastic flow of surgical activities, the model provides a bottom-up quantification of the energy footprint per patient, a metric largely absent in current healthcare modeling.

The remaining sections of this work are organized as follows. Section 2 examines the relevant literature on energy usage in healthcare facilities and the use of discrete-event simulation for hospital operations, emphasizing extant research gaps. Section 3 describes the suggested methodology, including the creation of the discrete-event simulation model, data sources, and the incorporation of energy-related variables. Section 4 presents the outcomes of the simulation studies, which include a baseline analysis and an evaluation of several organizational and technology energy efficiency measures. Section 5 reviews the findings and provides managerial perspectives, focusing on the implications for hospital decision-makers and sustainability-oriented planning. Finally, Section 6 summarizes the article and suggests areas for future investigation.

## 2. Literature Review

This section presents the current state of the art in the use of modeling and simulation approaches to promote energy efficiency and sustainability in healthcare facilities. The literature review has two goals: first, to examine how simulation-based methods have been used in healthcare settings and whether energy-related aspects are explicitly considered; and second, to identify methodological limitations and research gaps that motivate the current study.

### 2.1. Review Procedure

To carefully explore existing studies, a structured literature search was conducted utilizing the Scopus database. The review was led by three research questions. It should be noted that this review follows a structured rather than fully systematic approach; the broad search scope is intentional, as appropriate for an exploratory scoping study aimed at mapping an emerging research area.

1. Which modeling and simulation approaches are most commonly used to depict and enhance energy efficiency in healthcare facilities, and in what operational or structural contexts are they implemented?
2. How are energy characteristics, temporal dynamics, and performance indicators represented and incorporated into healthcare simulation models to evaluate sustainability?
3. To what extent do simulation models integrate energy-related objectives with operational and infrastructure constraints to aid decision-making that considers efficiency, sustainability, and quality of care?

The original search addressed the intersection of simulation and healthcare systems, yielding 19,345 documents. An organized combination of keywords was employed, divided into three theme areas: healthcare systems, decision-making and optimization, and simulation methodologies (Table 1).

**Figure 1:**
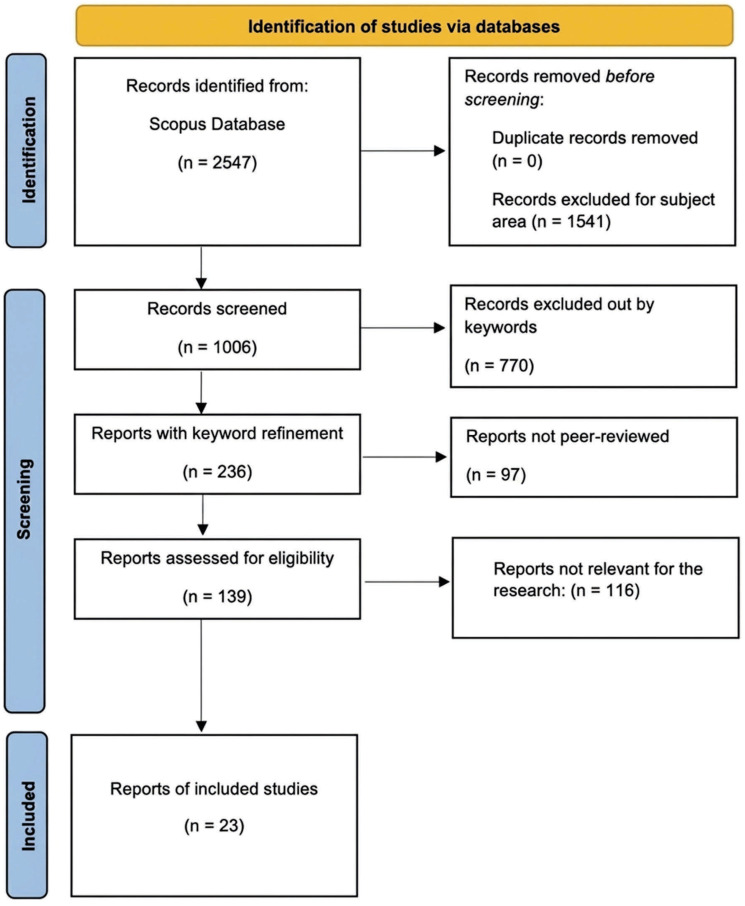
Structured search and selection flowchart.

To correlate the results with the study’s aims, a multi-step filtering technique was used (Figure 1). First, the search was limited to articles that directly mentioned healthcare systems, resulting in 2547 documents. Second, papers designated as solely medical or clinical subject areas were eliminated, removing biological studies unrelated to system-level modeling and lowering the dataset to 1006 entries. Further refining based on keywords relating to decision-making, efficiency, optimization, and simulation models, combined with a filter for peer-reviewed journal articles and reviews only, yielded 139 results. An abstract screening was then conducted to identify research that specifically addressed energy efficiency or sustainability at the system level. During this phase, 87 papers were discarded because they dealt with misaligned themes such as infectious illness modeling, patient monitoring technology, or logistics studies that addressed operational efficiency without taking energy into account. The remaining 52 articles underwent full-text analysis to establish thematic relevance and remove duplicates, yielding a final sample of 23 documents for the qualitative synthesis.

To guide the analysis and maintain consistency across investigations, an interpretive analytical framework was created (Table 2). The framework is organized into three dimensions: (i) modeling and simulation methodologies, (ii) energy representation, and (iii) objectives, restrictions, and energy indicators. The first component, Modeling and Simulation Techniques, looked at the most used paradigms (such as DES or agent-based modeling), their application contexts (e.g., emergency rooms, logistics), and whether these models were coupled with optimization techniques. The second dimension, Energy Representation, examined how healthcare facilities’ energy performance is represented in the models. It especially evaluated whether energy-related factors (for example, electricity use) were included, if temporal dynamics associated with energy use were modeled, and what performance criteria were used. Finally, the third analytical component addressed Objectives, Constraints, and Energy Indicators. It investigated whether simulation systems include objective functions associated with energy-related goals, if environmental or infrastructure constraints are considered, and whether energy KPIs are used to evaluate performance. This viewpoint evaluates whether simulation provides concrete support for sustainability-focused decision-making.

### 2.2. Results of the Literature Analysis

The analysis of the selected papers synthesizes the current state of simulation modeling in healthcare, reflecting the operational and environmental trends discussed in previous sections. The review confirms that while simulation is a mature instrument for operational efficiency, its application to environmental sustainability is still emerging and unevenly distributed.

Discrete-event simulation (DES) clearly remains the primary standard for optimizing clinical workflows. Several studies, such as [15,16], utilize this approach to resolve bottlenecks in Emergency Departments, while others, including [17,18], focus specifically on improving scheduling and team dynamics in Operating Rooms. Beyond single-unit optimization, the literature highlights a trend toward broader, strategic applications: for instance [19,20] propose hybrid and metamodeling approaches to manage complex system interactions, whereas [21,22,23] develop frameworks to support long-term resource allocation and value-based healthcare planning.

Regarding sustainability, the examination of energy relevance reveals a clear dichotomy between digital and physical domains. The integration of energy variables is predominantly found in studies focusing on ICT infrastructures. A significant cluster of research applies simulation-optimization to minimize energy consumption in digital ecosystems: for instance [24,25] optimize IoT and Cloud resource provisioning, while a broader group including [26,27,28,29,30] focuses on energy-aware architectures for edge computing, WBANs, and IoHT systems.

In contrast, models addressing the energy footprint of the hospital building itself are significantly fewer. Benchmarks and consumption analyses are provided by [6,31], while specific technological or structural interventions, such as HVAC automation and isolation room airflow, are examined in [32,33,34]. Broader environmental metrics remain niche, with [35,36] being the sole examples addressing sustainable water management and Life Cycle Assessment (LCA), respectively.

The fragmentation of sustainability integration is especially apparent when studying the essential components of simulation and simulation-optimization models:

1. Objective functions. The primary goals remain operationally driven, with most studies aiming to minimize waiting times or maximize throughput [15,16,17]. Purely energy-oriented objectives are standard in the digital domain [24,25] but are only emerging in broader hospital management frameworks.
2. Constraints. The feasibility of solutions is largely dictated by structural and operational limits [21,22]. While technological constraints are common in IT-focused studies [26], environmental constraints such as thermal comfort are less frequently applied [33,34].
3. Energy performance indicators. The transition to environmental KPIs is in its early stages. While digital studies explicitly measure energy metrics like network lifetime [28,29], indicators for physical sustainability remain rare, appearing only in specialized contributions [31,36].

In conclusion, the literature analysis confirms that while the methodological foundation for healthcare simulation is solid, a significant gap exists in applying these tools to the physical energy performance of hospital facilities.

### 2.3. Research Gaps

The literature review identifies numerous significant gaps, which motivate the current investigation. The principal novelty of this work resides in its integration of clinical stochasticity with physical energy consumption, a relationship that remained unexplored in all 23 studies identified in our structured literature search. Although prior DES-based studies have demonstrated strong performance in evaluating operational metrics, they have not modeled device-specific energy states as a direct function of clinical workflow dynamics.

First, despite the methodological maturity of simulation and optimization techniques in healthcare, most models continue to prioritize short-term operational efficiency, addressing localized constraints without explicitly tying them to environmental performance or sustainability goals. A key shortcoming is the insufficient incorporation of the physical and environmental components of healthcare facilities into simulation frameworks. This limitation is especially important because hospitals are among the most energy-intensive public buildings [6], with surgical rooms accounting for roughly 20–40% of overall hospital energy usage [37].

Nonetheless, simulation studies in surgical contexts mostly focus on throughput, scheduling, and delay reduction, with energy measurements rarely incorporated into the modeling logic. Even recent studies that show operational gains from advanced scheduling algorithms [34] typically include detailed energy KPIs.

Furthermore, the examined research indicates a poor relationship between energy factors and the spatial, organizational, and functional structure of hospital environments. Most contributions use a micro-level approach (e.g., single operating rooms or departments), while system-wide or pathway-oriented approaches are limited. When energy is included, it is frequently represented by aggregated building-level indicators or IT-related measures, rather than specifically modeling energy flows, temporal dynamics, or sustainability goals associated with healthcare procedures.

These shortcomings highlight the need for integrated simulation models that can capture clinical workflows, operational restrictions, and energy usage trends simultaneously. Embedding energy-related objectives, constraints, and performance indicators within discrete-event simulation frameworks is a promising approach to decision-making that improves both operational performance and environmental sustainability, one that this study aims to advance. To synthesize the main shortcomings identified in the literature and clearly position the contribution of this study, Table 3 presents a structured gap mapping that contrasts existing research practices with the expected advancements and specific contributions of the current study across key analytical dimensions.

## 3. Methodology

The research methodology employs a systematic and sequential strategy to integrate operational and energy viewpoints within a single simulation environment. The methodology is divided into four major phases: (i) problem identification and system boundaries, (ii) data collection and parameterization, (iii) simulation model creation, and (iv) results evaluation.

The first phase concentrated on discovering energy inefficiencies in healthcare facilities, with a special emphasis on operating rooms, which have high energy intensity and complicated organizational dynamics. This phase was aided by a comprehensive literature study aiming at identifying methodological shortcomings in the incorporation of energy measurements into healthcare simulation models.

The second phase included data collection and the creation of a comprehensive input database. The database contains process descriptions, resource limitations, activity durations, patient flows, and energy consumption profiles. Data were collected using a hybrid strategy that included scientific literature, clinical information, and expert-based estimates, assuring both realism and consistency with real-world operations.

The third phase involved creating a simulation model and outlining the cause-and-effect links between clinical activities, resource use, and energy consumption. The Discrete-Event Simulation approach was chosen because it is well-suited for modeling complex systems with stochastic behavior, limited resources, and time-dependent patient flows. DES allows healthcare processes to be represented as discrete events unfolding across time, reflecting both variability and operational restrictions. Examples include patient arrivals, diagnostic imaging examinations, and surgical operations.

The final phase centered on model execution and results evaluation. The simulation outputs were assessed to evaluate operational performance and energy usage patterns, as well as to identify potential organizational and technological improvements.

### 3.1. System Boundaries

The system boundaries were determined at the micro level, with an emphasis on orthopedic surgery rooms, which were chosen for their high energy demand and administrative relevance. Orthopedic surgery was selected for its high energy intensity, driven by the extensive use of power devices, imaging equipment, and sterilization processes, making it a particularly relevant context for an energy-conscious analysis. The focus on elective procedures further supports this choice, as scheduled interventions allow for a more tractable modeling approach with predictable patient flows and resource allocation. The configuration of two operating rooms and one diagnostic imaging unit reflects a realistic and representative micro-level layout, sufficiently complex to capture the interactions between clinical workflows and energy consumption while remaining manageable for simulation purposes. Importantly, different layout configurations, varying in the number of rooms or diagnostic units, would naturally entail different patient volumes and arrival patterns; however, since demand and service capacity are intrinsically linked, the structural relationships modeled here retain their validity across configurations, making this case study representative of a broader class of hospital micro-systems. To represent the interdependence of clinical efficiency and energy usage, the simulation scope was divided into two logically connected functional units: the Diagnostic Imaging Unit and the Orthopedic Surgery Unit. The Diagnostic Imaging Unit is responsible for all preoperative diagnostic imaging activities, including patient registration, specialist consultations, and diagnostic imaging studies, in particular X-ray and Magnetic Resonance Imaging (MRI). The modeled pathway encompasses the complete process, from patient arrival to diagnostic imaging procedure completion, and includes both human resources and high-energy-consuming medical equipment. The Orthopedic Surgical Unit includes the entire perioperative pathway (see Appendix A). The process is divided into three phases: preoperative (admission, bed assignment, and surgical eligibility checks), intraoperative (patient transfer to the operating room, anesthesia, and surgical execution), and postoperative (patient monitoring in the PACU until transfer to the ward).

Within these parameters, logistical support operations such as operating room sanitization and equipment cleaning were precisely modeled. These transition times are crucial from an energy standpoint, as infrastructure-based loads (such as HVAC and lighting systems) continue to operate despite the absence of clinical activity. Processes outside of the operating block, such as long-term inpatient stays, were omitted from thorough modeling, yet logical interfaces were preserved to accommodate downstream capacity restrictions.

### 3.2. Input Data

The simulation model was parameterized with a hybrid data collection technique that combined secondary data (i.e., literature-based values, clinical databases) and primary data based on expert interviews. This method allows the specification of activity durations, resource availability, patient route probabilities, and energy consumption characteristics. A dedicated dataset was created for 57 elective orthopedic surgeries, which were categorized by anatomical district and difficulty degree. To preserve the stochastic nature of real clinical workflows and represent case-mix variation, activity durations were not modeled deterministically. Instead, empirical mean values and standard deviations (see Appendix B) were converted into probability distributions (triangular and log-normal) based on the characteristics of each process phase, such as patient preparation, surgery, and recuperation. The selection of each distribution type was guided by the statistical properties of the underlying activity. Log-normal distributions were adopted for surgical durations, which typically exhibit right-skewed variability consistent with occasional prolonged procedures. Triangular distributions were applied to preparation and recovery phases, for which expert interviews provided reliable estimates of minimum, most likely, and maximum durations, making this distribution the most appropriate choice given the available data. Routing probabilities were defined on an empirical basis, combining structured interviews with clinical experts and a review of the surgical case mix for the selected elective orthopedic procedures. The 80/20 split between X-ray and MRI reflects the observed prevalence of X-ray in routine pre- and postoperative diagnostic checks, with MRI reserved for cases requiring more detailed soft tissue assessment. Energy consumption was determined by multiplying simulated operation periods by each resource’s nominal power rating (see Appendix C). The model distinguishes between two types of energy consumption: infrastructure-based loads (e.g., HVAC systems and lighting) and activity-dependent variable loads (e.g., medical equipment like MRI scanners, C-arms, and arthroscopy towers) that vary based on usage and duty cycles.

**Figure 2:**
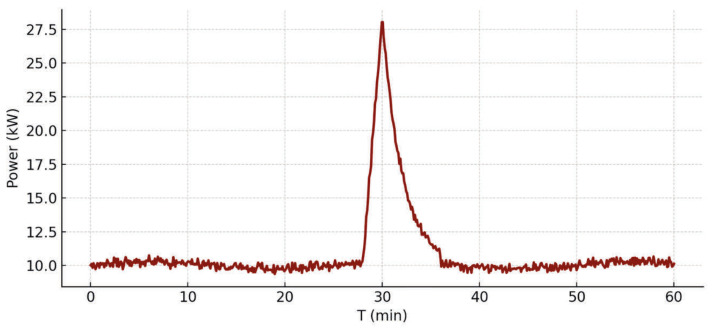
Example of a peak in a load profile.

Medical equipment was modeled in three operational states (idle, base load, and peak), allowing the simulation to capture intermittent power requirements and realistic load profiles (Figure 2).

### 3.3. Simulation Model

To enable model development and validation, the simulation model was constructed in FlexSim Healthcare (Version 25.2.0, FlexSim Software Products Inc., Orem, UT, USA), which has a dedicated healthcare object library and three-dimensional visualization capabilities. The DES paradigm brings together the Diagnostic Imaging Unit and the Orthopedic Surgical Unit into a single framework.

The simulation was set up to simulate a typical operational day, from 8:00 a.m. to 8:00 p.m., with a 45-min non-operational break. The outputs of each replication were averaged to derive mean performance indicators. The standard deviation across replications was computed to assess the stability of results, ensuring that reported values reflect the expected system behavior rather than the outcome of a single simulation run. To establish statistical robustness and account for the stochastic variability inherent in the model, fifty independent replications of the daily operational schedule were carried out. For each replication, the simulation generated a complete set of performance indicators, including resource utilization rates, patient wait times, and throughput. Mean values were then computed across all replications and used as the primary basis for result reporting. Standard deviations were calculated to assess output stability and are reported alongside mean values for the key operational indicators. Energy consumption figures were subsequently derived deterministically by applying the power profiles of each device to the mean simulated activity durations. Because of this deterministic derivation, the energy and emission values reported in Table 4 do not carry confidence intervals and should be interpreted as indicative central estimates, consistent with an exploratory modeling approach.

#### 3.3.1. Diagnostic Imaging Unit

**Figure 3:**
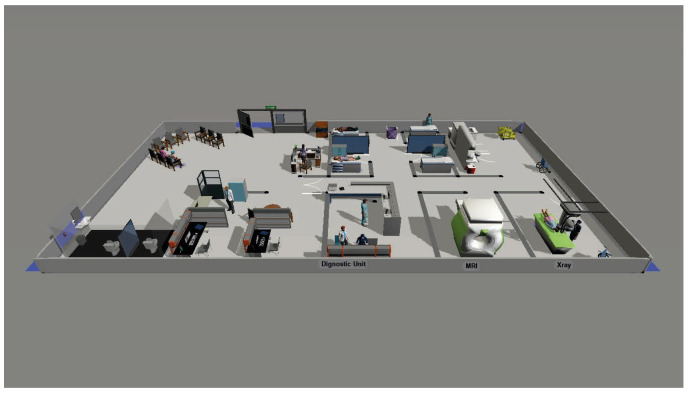
Representation of the diagnostic unit in the FlexSim environment.

The Diagnostic Imaging Unit paradigm encompasses the entire preoperative imaging route. The physical layout consists of a reception area, waiting room, examination rooms, and two dedicated diagnostic suites: one MRI room and one X-ray room (Figure 3).

- Technological resources. Energy-intensive diagnostic equipment (MRI and X-ray) was explicitly modeled. Patient routing to imaging modalities is probabilistic and is determined by the surgical procedure type.
- Staff. The unit consists of a receptionist, two nurses, two radiologists, and two radiographers who perform diagnostic imaging examinations.

#### 3.3.2. Orthopedic Surgical Unit

**Figure 4:**
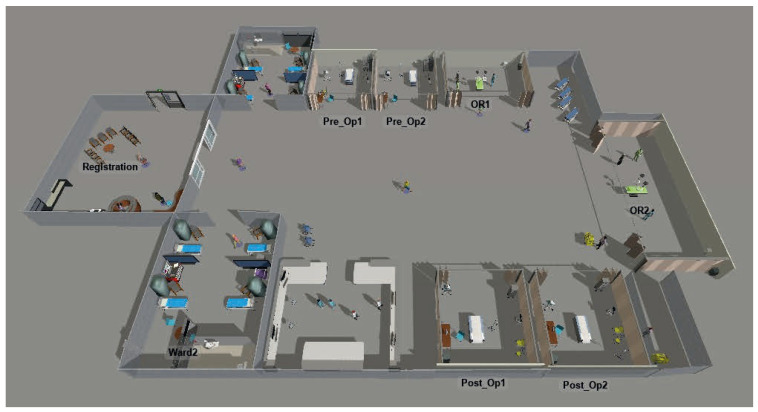
Representation of the orthopedic surgical unit in the FlexSim environment.

The Orthopedic Surgical Unit replicates the perioperative process for elective procedures (Figure 4). The modeled layout includes a reception area, an eight-bed ward, two preoperative preparation stations, two operating rooms, and two post-operative recovery stations.

- Technological resources. Operating rooms are outfitted with procedure-specific medical instruments. The probability of use for each intervention, as well as the simulated surgical case mix, determines device activation. Multiparameter monitors, infusion pumps, and motorized beds are examples of additional equipment used throughout the pre- and postoperative stages.
- Staff. Administrative staff, ward nurses, PACU nurses, preoperative physicians, and dedicated OR teams (surgeons, anesthetists, scrub nurses) make up the workforce. Two operators are allocated to the sanitization and sterilization chores, which are critical for OR turnover.

#### 3.3.3. Energy Consumption Model

The total daily energy consumption of the operating block ($E_{System}^{Day}$) is calculated as the sum of the energy consumed by the diagnostic and the orthopedic surgical units.

(1) $$E_{System}^{Day}=E_{Diagn}^{Day}+E_{OR}^{Day}$$

The daily energy consumption of unit x($E_{x}^{Day}$, in kWh) is computed by aggregating the consumption of each device d across its discrete operational states j∈J and all simulated patients N:

(2) $$E_{Unit}^{Day}=\sum_{d}\sum_{j\;\in \;J}P_{j,d}\cdot \left(\sum_{i=1}^{N}\frac{T_{i,j,d}}{60}\right)$$

where:

- *d* is the index running over all devices considered in the unit.
- *J* is the set of discrete operational states of device *d*, defined as *J* = {Idle, Base Load, Peak}.
- $P_{j,d}$ is the average power drawn by device d in state *j* (in kW).
- *N* is the total number of simulated surgical interventions or patients per day.
- $T_{i,j,d}$ is the duration in minutes that device d is in state *j* during intervention *i*.

The energy modeling framework establishes that the duration of stay in each operational state ($T_{i,j,d})\;$ is determined by the specific simulation logic of the functional unit. To ensure physical consistency and accurate energy accounting, the model applies a time decomposition logic: the total operational duration allocated for the device $\left(T_{i,d}^{Op}\right)$ is strictly portioned in the Total Active Time $\left(T_{i,d}^{act}\right)$, representing the effective usage, and the Idle Time $\left(T_{i,d}^{idle}\right)$, representing the standby period. This principle ensures that no operational time remains unaccounted for and is applied consistently across units, even if using two different calculation methods:

1. Surgical Unit Logic: since intra-operative usage is intermittent, operational durations are derived probabilistically based on the procedure duration $T_{i}$ and the probability of usage *p*$\left(s_{i\;},\;d\right)$ of device *d* for the specific surgery type $s_{i}$.
  The implementation utilizes the Average Duty Cycle δd to apply the partition constraint
  - Active Time $\left(T_{i,d}^{act}\right)$: calculated as the portion of the procedure where the device is effectively running.
    (3) $$T_{i,d}^{act}=T_{i}\cdot \;p\left(s_{i\;},\;d\right)\cdot \delta d$$
  - Idle Time $\left(T_{i,d}^{idle}\right)$: calculated as the remaining time where the device is present but in standby.
    (4) $$T_{i,d}^{idle}=T_{i}\cdot \;p\left(s_{i\;},\;d\right)\cdot (1-\delta d)$$
2. Diagnostic imaging Unit Logic: The durations $\left(T_{i,d}^{Op}\right)$ are determined by the probabilistic patient routing and the fixed duration of maintenance activities between patients, ensuring that actual utilization and idle times reflect the operational constraints of the diagnostic imaging suite.
  The calculation is further refined by partitioning active time into base and peak components, a necessity for accurate energy measurement, using the partition factor ($\beta_{d})$:
  (5) $$T_{i,d}^{base}=T_{i,d}^{act}\cdot \beta_{d}\;\mathrm{and}\;T_{i,d}^{peak}=T_{i,d}^{act}\cdot \left(1-\beta d\right)$$

## 4. Results

In this section, the results of the discrete-event simulation are presented in detail, starting from the baseline “AS-IS” scenario and subsequently evaluating the effects of targeted organizational and technological interventions designed to improve both operational performance and energy efficiency.

### 4.1. AS-IS Results

The examination of the discrete-event simulation models for the Diagnostic imaging and Orthopedic Surgical Units under the “AS-IS” scenario establishes a solid foundation for future improvement evaluations. The results show a strong relationship between workflow efficiency and energy intensity per patient, with space limits highlighted as the key system bottlenecks.

**Figure 5:**
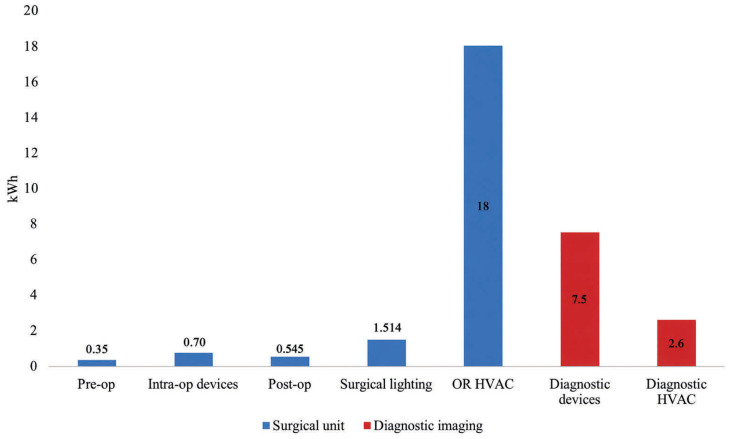
Breakdown of the energy consumption per patient in the different phases of the simulation.

- Diagnostic imaging unit. In the baseline situation, with 30 patients per day and routing set to 80% for X-ray and 20% for MRI, energy consumption and equipment use showed large asymmetries.
  o  X-ray: Each examination consumes about 1.1 kWh per patient (26.3 kWh/day), which increases to 2.1 kWh per patient when the assigned HVAC share is considered. Despite frequent use, the X-ray machine is idle for 55.6% ± 4.1% of the time, with a base load of 33.6% ± 3.2% and a peak load of only 1.04% ± 0.10%.
  o  MRI: MRI energy use is significantly greater, at 33.1 kWh per patient each test (41.1 kWh with allocated HVAC). The MRI is idle for 69.6% ± 10.3% of its operating period, which reflects the need for continual magnet cooling, with a base load of 20.6% ± 7.6% and a peak of 6.9% ± 2.5%.
- The Diagnostic Imaging Unit’s weighted average energy consumption is 10.1 kWh per patient. The analysis of patient waiting dynamics shows that the majority of delays are caused by a lack of diagnostic imaging room availability (“Wait for Location” state), demonstrating that throughput is mostly limited by structural capacity.
- Orthopedic Surgery Unit. The simulation of six elective operations over two operating rooms emphasizes the importance of space restrictions in workflow and energy intensity. Patient wait times are dominated by location unavailability (“Wait for Location” state), with an average of 56.52 min, compared to 26.51 min for personnel availability (“Wait for Staff” state). Operating rooms are used 36.91% ± 5.8% of the time, with sanitization and preparation (Maintenance) taking up an additional 20.97% ± 1.4%. The remaining 41.78% ± 5.9% represents idle time. The total energy requirement for each surgery patient, including fixed and activity-dependent loads, is 21.1 kWh. The breakdown by component reveals the dominance of HVAC fixed loads (18 kWh per patient), making energy intensity extremely sensitive to room downtime. Surgical lighting contributes to 1.514 kWh per patient, while intraoperative devices account for 0.70 kWh, with the C-arm responsible for 75.5% of device consumption. The pre- and postoperative phases contribute 0.35 and 0.545 kWh, respectively (Figure 5).

The validation and comparison of results confirm the consistency of the simulation model with theoretical estimates, as the values obtained for the surgical unit are in line with the expected order of magnitude of 18 kWh per patient, derived from the analytical assessment of the cumulative energy demand of HVAC systems, lighting, and biomedical devices. The intraoperative phase is confirmed as the dominant energy component in both analytical approaches.

The AS-IS scenario indicates that operational inefficiencies, particularly long rotation times, increase energy costs per patient due to fixed infrastructural loads, laying the groundwork for developing targeted improvement scenarios.

### 4.2. Improvements Proposal

The AS-IS analysis highlighted two key causes of high energy intensity: fixed infrastructural loads dominated by HVAC and operational inefficiencies due to space turnover. Three improvement scenarios were developed and simulated, combining organizational strategies to increase operating room throughput and reduce idle consumption in diagnostic imaging, together with technological interventions aimed at lowering nominal energy demand.

The first intervention focused on organizational optimization within the Operating Room to leverage HVAC dilution. Given that HVAC is the most energy-intensive component (18 kWh per patient), boosting operating throughput effectively reduces its per-patient impact. The simulated intervention raised daily procedures from six to eight (four in each OR) within the same 12-h shift by incorporating short, compatible elective surgeries. As a result, the HVAC contribution per patient dropped from 18 to around 13.5 kWh, and total intraoperative energy usage decreased from 21.1 kWh to 16.6 kWh per patient, demonstrating that boosting operating room use is a successful technique for reducing fixed-load energy consumption. It should be noted that per-patient energy intensity improvements do not necessarily translate into reductions in absolute daily emissions when throughput increases simultaneously. In this scenario, the daily CO_2_ rises from 32.4 to 33.6 kg/day because more patients are treated, even though each patient’s individual footprint decreases.

In parallel, a technological intervention was evaluated regarding the lighting systems. This scenario assessed the impact of replacing standard surgical lamps (1.0 kW) with LED lighting systems. The modification lowered lighting consumption from 1.5 kWh to around 0.2 kWh per patient, resulting in a direct savings of 1.3 kWh per procedure. Additionally, the reduced heat dissipation from LEDs lowers the internal heat gain, further reducing the need for HVAC cooling.

Furthermore, a combined scenario integrating both scheduling optimization and LED upgrade was analyzed to evaluate potential synergies. The results show that this combination yields the most significant reduction, lowering energy intensity to 15.3 kWh per patient.

Finally, the optimization analysis was extended to the Diagnostic Imaging Unit to address MRI idle time. In this unit, energy consumption is mostly driven by extended idle time for magnet cooling [18,38]. The recommended strategy rearranged MRI scheduling, concentrating sessions on two active days per week and turning off the scanner on the remaining days. Consequently, the weekly MRI use decreased from 1125 kWh to 454 kWh, resulting in a 60% savings, and the average energy usage per patient in the unit was reduced from 10.1 to 8.5 kWh. The weekly baseline (1125 kWh) was obtained by scaling the daily model output across five operational days; under the bundled scenario, full active consumption applies to two days while the remaining three days incur stand-by-only loads, yielding the 454 kWh weekly estimate. The per-patient figure (10.1 to 8.5 kWh) reflects a separate calculation based on the average energy per examination on active days.

Table 4 summarizes the detailed results regarding energy consumption and estimated CO_2_ emissions for all simulated scenarios. CO_2_ emissions were estimated by applying the Italian national grid emission factor of 0.256 kg CO_2_/kWh to the simulated energy consumption values.

## 5. Discussion and Managerial Insight

The analysis conducted in this study indicates that energy efficiency and environmental sustainability in healthcare facilities are closely linked to workflow organization and operational decision-making. Rather than being determined primarily by the volume of clinical activity, energy consumption appears to be substantially shaped by the continuous availability of infrastructure and equipment. This finding confirms a well-established paradox of high-technology settings: energy demand is largely driven by fixed and quasi-static loads that persist independently of actual utilization levels.

As highlighted in the results section, the most effective way to reduce energy usage and CO_2_ emissions is through a combination of organizational and technological interventions. The combined scenario demonstrates that addressing fixed infrastructural loads through organizational measures (HVAC dilution) and nominal loads through technical upgrades (LED) provides a multiplier effect that outperforms the effects of individual actions.

When multiple strategies’ effectiveness is compared, a distinct hierarchy emerges. Organizational solutions consistently outperform solely technological ones in both surgical and diagnostic imaging settings. Increasing throughput and optimizing HVAC consumption in surgical units generates a significantly higher carbon saving compared to lighting upgrades alone. This effect is explained by the structural characteristics of operating rooms, which are typically 3 to 6 times more energy-intensive than the rest of the hospital due to strict air quality, temperature, and sterility standards [39]. Under these conditions, the marginal energy cost of an extra procedure is negligible, while increased usage dramatically reduces energy intensity per patient.

The impact of organizational strategies is considerably more noticeable in the diagnostic imaging domain. The MRI bundling scenario resulted in a drastic reduction in energy consumption. This finding emphasizes that, for energy-intensive systems with significant idle consumption, work organization and scheduling strategies are the most effective sustainability lever. This aligns with recent literature illustrating that reducing idle activity provides greater benefits than marginal efficiency gains in device performance.

Replacing conventional surgical lighting with LED lights results in a minor reduction in emissions (about 0.3 kg CO_2_ per patient). While this intervention is technically solid and helps to reduce nominal loads, it does not address underlying process inefficiencies and thus cannot compensate for long idle times or unused spaces.

Although this analysis focuses on Scope 2 emissions from electricity usage, the effects go beyond that. Improved operating room use can also help to lower Scope 1 emissions by avoiding the unwanted dispersion of volatile anesthetic gases caused by extended theater activation [7]. Similarly, Scope 3 emissions can be indirectly decreased by reducing cancellations, improving schedule dependability, and minimizing the waste of sterile materials and consumables [40].

From an economic standpoint the energy efficiency is not simply an environmental goal, but also a financial resilience plan. Hospitals can increase financial stability while confirming their sustainability pledges by reducing their reliance on volatile energy prices.

The results provide three crucial insights for managers:

1. Integrating operational and energy management. Clinical operations and facility management must be addressed together rather than as distinct domains. Decisions about scheduling, case mix planning, and resource allocation frequently have a higher impact on energy use than engineering interventions alone. Integrating energy data into operational planning processes is so critical.
2. Adoption of dynamic, process-oriented key performance indicators. Energy- and emission-based metrics, like kWh per patient or kgCO_2_ per procedure, should be used alongside traditional static indicators. Making the environmental cost of therapeutic activities apparent promotes more informed decision-making and the adoption of sustainable practices.
3. Priority is given to organizational intelligence. Before investing in capital-intensive technological retrofits, healthcare facilities may benefit from focusing on optimizing current resources through organizational redesign and schedule changes. These initiatives may offer faster returns on investment and potentially facilitate the effective adoption of future technology advancements. These insights are grounded in an exploratory single-site model and require validation against empirical operational data before generalization.

Overall, the discussion emphasizes that healthcare sustainability is essentially a managerial and organizational concern, necessitating the development of tools and models capable of addressing clinical performance, resource use, and environmental impact simultaneously.

A critical practical challenge in realizing these organizational gains lies in the structural fragmentation of hospital decision-making. Scheduling, clinical operations, and facility management typically function as separate domains, with limited data exchange and misaligned incentive structures. As a result, decisions that could jointly optimize patient throughput and energy consumption, such as concentrating MRI sessions or filling OR slots with compatible short procedures, are rarely coordinated across these functions.

To overcome this barrier, hospitals can pursue a few targeted actions. First, establishing a cross-functional governance structure, bringing together OR coordinators, anesthesiologists, radiologists, and facility managers, creates the institutional accountability needed to align scheduling decisions with energy performance goals. Within this structure, clearly defining which roles are responsible for monitoring energy KPIs and who holds authority over scheduling adjustments is essential to avoid the diffusion of responsibility that typically stalls sustainability initiatives. Second, embedding energy intensity metrics, such as kWh per patient or kgCO_2_ per procedure, into the same reporting dashboards used for operational performance makes energy visible within existing clinical governance frameworks, rather than relegating it to a separate sustainability report. Third, simulation tools such as the one developed in this study can play a concrete role in these settings: by quantifying the energy impact of scheduling alternatives, they provide a shared, evidence-based reference that supports negotiation across departments with different priorities.

## 6. Conclusions

The primary goal of this study was to close the gap between the operational management of healthcare procedures and the growing need for energy efficiency and environmental sustainability in hospital facilities. This study demonstrates how clinical workflows can be approximated within a simulation environment while also quantifying the energy consumption associated with healthcare operations through the development and implementation of an integrated discrete-event simulator. In doing so, the proposed framework broadens standard healthcare simulation beyond operational success indicators to include energy-related issues, which are becoming increasingly important for sustainable hospital management.

The findings demonstrate the effectiveness of simulation as a decision-support tool in complex healthcare settings. The model is designed to capture the unpredictable and resource-constrained nature of hospital procedures, allowing for the explicit measurement of energy use across diagnostic imaging and surgical pathways. A preliminary consistency check of the simulation architecture suggests that the model’s energy consumption estimates are broadly aligned with theoretical assumptions and order-of-magnitude values reported in the literature. While this does not constitute a formal validation against field-measured data, it supports the internal plausibility of the proposed approach and provides a reasonable basis for exploratory scenario analysis. As a result, the model offers hospital decision-makers a structured framework for exploring organizational and technical improvement scenarios while maintaining quality of care and operational continuity.

The principal limitation of this study lies in the nature of the input data. Owing to the absence of extensive metering infrastructures, several energy-related parameters were estimated on the basis of scientific literature and expert judgment rather than direct field measurements. Although this approach is suitable for exploratory modeling, it necessarily entails a degree of approximation. In addition, the scope of application was intentionally restricted to elective orthopedic surgery. While this delimitation enabled a more focused and in-depth analysis, it also required simplifying assumptions regarding interactions with other hospital departments and shared resources, which are inherently interdependent in real-world hospital operations.

Notwithstanding these limitations, the findings of this study provide a foundation for several future lines of research aimed at overcoming the current constraints. First, the integration of the simulation framework with real-world energy data, acquired through sensors, smart meters, or hospital energy management systems, represents a necessary step toward enhancing both model accuracy and empirical validity. Second, future developments should incorporate multi-objective optimization approaches capable of balancing clinical performance, energy consumption, and environmental impact through the application of advanced algorithms and sensitivity analyses. Third, extending the proposed framework to additional hospital departments and clinical pathways will be essential for evaluating its scalability and for supporting the development of a hospital-wide, energy-aware decision-support system.

## Figures and Tables

**Table 1:** Overview of keywords used in the literature search.

Research Area		Keywords	
	Healthcare system	“Healthcare system”	
AND	Decision & Optimization	“Decision making”	
		OR	“Optimization”
		OR	“Decision support system”
		OR	“Efficiency”
AND	Simulation approach	“Simulation”	
		OR	“Queueing theory”

**Table 2:** Analytical framework for the literature review.

Analytical Framework		
Modeling and Simulation Techniques	Energy Representation	Objectives, Constraints and Energy Indicators
Types of simulation models Application domains Integration with optimization algorithms	Inclusion of energy parameters Modelling of temporal energy dynamics Use of energy performance metrics	Definition of sustainable objective functions Environmental and infrastructure constraints Use of energy-related KPI for performance evaluation

**Table 3:** Summary mapping of the research gaps identified in the literature review.

Gap Mapping			
Dimension	Literature Provides	Expected	Contribution of the Study
Modelling Focus	Operational performance, patient flow, waiting time reduction	Integration of energy efficiency in core clinical models	Shift from purely operational DES to Sustainability-Driven DES, linking clinical stochasticity with physical energy flows.
Constraints and KPIs	Staff availability, resource use, patient throughput	Standardized environmental constraints and energy-related KPIs for decision-making	Identification and implementation of energy-aware constraints and indicators in OR management
Scope and Level	Focus on micro-level operations, but limited to clinical or logistic aspects	Multidimensional models addressing both operational and environmental targets at the micro level	Integrated evaluation of clinical performance and environmental sustainability in surgical scheduling
Energy Representation	Sparse and often limited to IT infrastructure or general building-level metrics	Explicit modelling of energy flows, temporal dynamics, and sustainability targets in healthcare processes	Inclusion of energy parameters and metrics directly related to OR usage and scheduling decisions

**Table 4:** Estimated CO_2_ emissions per patient and per day in baseline and alternative scenarios.

Scenario	Energy (kWh/Patient)	CO_2_ (kg/Patient)	Patients/Day	CO_2_ (kg/Day)
Surgery baseline	21.1	5.4	6	32.4
Surgery HVAC	16.6	4.2	8	33.6
Surgery LED	19.8	5.1	6	30.6
Surgery HVAC + LED	15.3	3.9	8	31.2
Diagnostic Imaging baseline	10.1	2.6	30	78
Diagnostic Imaging scenario	8.5	2.2	30	66

**Figure A1:**
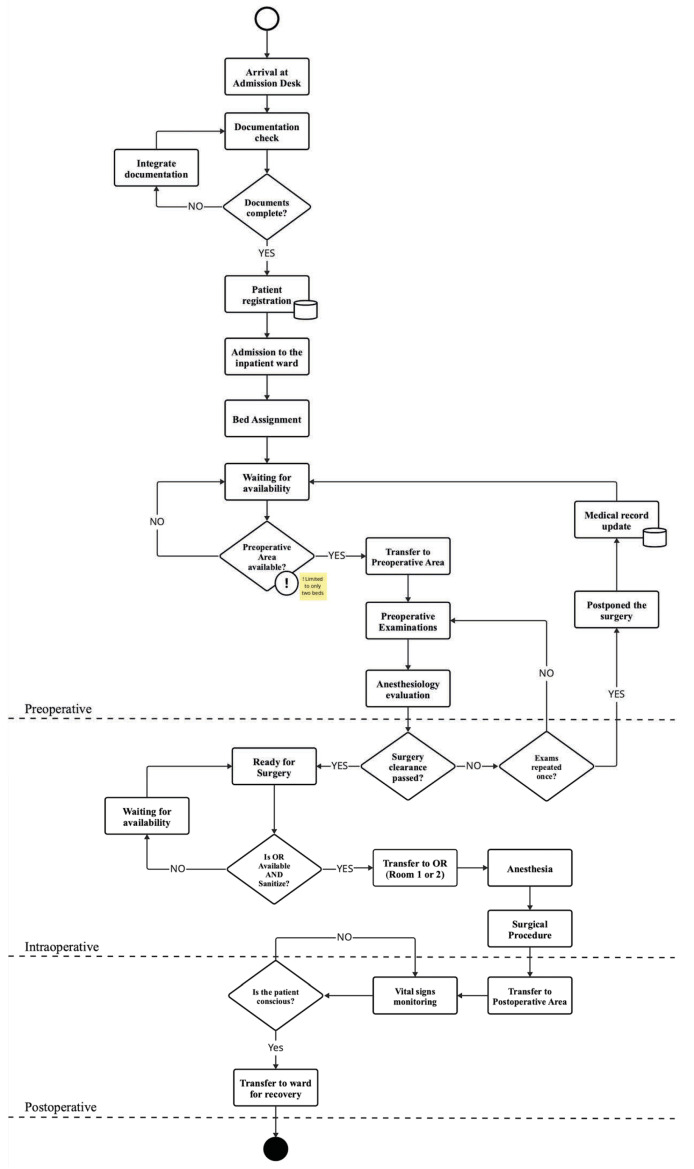
Orthopedic surgical process flow divided into preoperative, intraoperative and postoperative phases.

## Data Availability

The raw data supporting the conclusions of this article will be made available by the authors on request, since the data used as input for the simulation includes data that combines clinical activity data, scientific literature, and expert judgment contributions: The full data and settings of the input for the Flexsim Healthcare simulation will be made available by the authors on request.

## Abbreviations

The following abbreviations are used in this manuscript:

MRI	Magnetic Resonance Imaging
LED	Light Emitting Diode
OR	Operating Room
HVAC	Heating, Ventilation and Air Conditioning
DES	Discrete Event Simulation
KPI	Key Performance Indicator
IoT	Internet of Things
IT	Information Technology
PACU	Post-Anesthesia Care Unit
WBAN	Wireless Body Area Network
LCA	Life Cycle Assessment

## Appendix B

This appendix provides a detailed temporal analysis of the procedures. The data regarding intervention durations is aggregated by anatomical district and complexity. Additionally, the tables outline the specific operating times for the devices and the duration of each distinct process phase. The values reported represent simulation input parameters derived from a hybrid data collection approach combining scientific literature, structured interviews with clinical experts. Average durations and standard deviations are used as input parameters to define the probability distribution adopted in the simulation model.

**Table A1:** Elective orthopedic surgeries aggregated by anatomical district and complexity level, based on duration.

Anatomical District	Surgical Complexity Level	Type of Surgeries	Average Duration (min)	Average Standard Deviation (min)	Average Buffer Time (min)
Hip	Medium	1	90	22.5	45
	High	1	150	45	90
Lower Limb	Medium	1	120	33.6	70
Upper Limb	Low	1	55	9.4	20
Pelvis	Medium	1	100	30	60
Ankle	Low	1	50	16.5	35
	Medium	4	85	20.3	43.8
Spine	Low	1	50	14	30
	Medium	5	87	16.8	51
	High	3	143.3	29.4	88.3
Fingers	low	1	40	11.2	25
Femurs	Medium	1	90	25.2	55
Knee	Low	1	35	8.8	20
	Medium	4	88.8	21	43.8
	High	1	130	36.4	75
Elbow	High	1	140	38.8	65
Hand	Low	4	30	14.7	18.8
	Medium	3	75	15.1	36.7
Foot	Low	4	38.8	10.8	25
	Medium	7	72.9	19.5	40
Wrist	Medium	3	106.7	29.9	63.3
Shoulder	Medium	3	108.3	27.1	55
	High	1	135	28.2	60
Tibia	Medium	1	85	23.8	50
Miscellaneous	Low	1	30	9.9	20
	Medium	1	60	16.8	35
	High	1	130	28.6	60

**Table A2:** Macro-activities durations in the orthopedic surgical pathway.

Phase	Activity	Average Duration (min)	Average Standard Deviation (min)	Variability Factors
Diagnostic and preparatory tests	X-ray imaging	10	3	Minor variability due to exam execution
	CT imaging	15	5	Moderate variability
	MRI imaging	30	10	Higher variability, longer exam
	Laboratory tests (blood, urine)	12	4	Variability from sampling procedures
	Specialist consultations	25	10	Depends on patient complexity
Admission and registration	Arrival at hospital	15	5	Variability due to patient flow
	Documentation check and integration	15	5	Delays if incomplete
	Patient registration and bed assignment	25	10	Dependent on ward capacity
Preoperative preparation	Transfer to pre-operative area	10	3	Possible bottlenecks
	Anaesthesiology evaluation	20	5	Longer in case of comorbidities
	Surgery clearance check	5	1	Final eligibility verification
Intraoperative	OR preparation and patient transfer	30	6	Dependent on turnover and logistical efficiency
	Anaesthesia induction	20	8	Technique-dependent
Postoperative recovery	Vital signs monitoring	120	40	Standard ~2h, prolonged if complications
	Transfer to ward	30	10	Dependent on bed availability
Ward stay	Ward monitoring	1440 (24 h)	720	Typically 1–3 days
Follow-up	Control imaging	20	7	Outcome verification
	Post-op laboratory tests	10	4	Routine postoperative blood analysis
	Clinical control visit	15	5	Routine evaluation
Turnover & sterilisation	Instrument reprocessing	50	15	Depends on sterilisation workload and turnover

## Appendix C

This appendix presents detailed tables outlining the technical specifications of the devices employed. Specifically, the data includes the power ratings and the respective duty cycle for each component and phase. Note that the parameters listed document the full clinical pathway context; the simulation model itself was scoped to MRI and X-ray only. Parameters for CT and other modalities are provided for completeness and to support potential future extensions of the model.

**Table A3:** Estimated power demand of infrastructural systems that remain constantly active.

Resource	Operating Mode	Power Range (W)	Duty Cycle (%)	Notes
HVAC (OR)	Active	3000–6000	100	Depends on air changes per hour (ACH) and thermal set-points
Surgical lighting	Active	500–1500	100	Target lux maintained throughout OR use
Anaesthesia workstation	Standby	100–200	100	Continuous readiness and monitoring
Multiparameter monitoring	Active	50–100	100	Small but constant load

**Table A4:** Estimated power demand of clinical devices active only during specific phases.

Resource	Operating Mode	Power Range (W)	Duty Cycle (%)	Notes
Electrosurgical unit (ESU)	Active	300–800	5–20	Pulsed activation, low average duty
Arthroscopy tower	Active	500–900	30–80	Includes light source, pump, and camera
C-arm fluoroscopy	Active	2000–5000	1–10	Short pulses; high instantaneous draw
Suction	Active	100–300	20–60	Varies with surgical technique and field conditions
Infusion pump	Active	50–100	40–100	Low draw potentially continuous
Orthopaedic drill	Active	80–200	5–30	Intermittent use during drilling
Oscillating bone saw	Active	100–300	5–20	Short bursts during bone cutting
Surgical microscope	Active/Standby	200–500	20–100	Includes lighting and electronics
Irrigation pump	Active	50–200	20–80	For arthroscopy or wound irrigation
Powered screwdriver	Active	50–150	10–50	Short cycles during screw/pin insertion

**Table A5:** Estimated power demand of diagnostic imaging and recovery equipment.

Phase	Resource	Power Range (W)	Duty Cycle (%)	Notes
Diagnostic and preparatory tests	X-ray generator	20,000–50,000	1	Very short exposure high instantaneous draw
	CT scanner	30,000–60,000	5	High draw only during acquisition
	MRI	15,000–30,000	10–40	Active load during image acquisition
	Ultrasound	100–500	10–60	Regional anaesthesia, vascular access, focused checks
	Diagnostic devices	50–200	5–20	ECG, spirometer, blood analysers
Postoperative recovery and follow-up	Multiparameter monitor	50–100	100	Bedside monitoring throughout recovery
	Infusion pump	50–100	40–100	Potentially continuous
	Motorized bed	200–400	10	Short activations for positioning
	Oxygen therapy system	100–500	60–100	Flow generators, humidifiers
	Post-operative X-ray	20,000–50,000	1	Control imaging if clinically required
	Ultrasound (checks)	100–500	10–60	Focused post-op assessments

## References

[1] United Nations Regional Information Centre (UNRIC) (2019). Causes of Climate Change.

[2] McKinsey & Company (2022). The Net-Zero Transition: What It Would Cost, What It Could Bring. McKinsey Sustainability Insights. https://www.mckinsey.com/capabilities/sustainability/our-insights/the-net-zero-transition-what-it-would-cost-what-it-could-bring.

[3] Pichler P.P., Jaccard I.S., Weisz U., Weisz H. (2019). International comparison of health care carbon footprints. Environ. Res. Lett..

[4] Lenzen M., Malik A., Li M., Fry J., Weisz H., Pichler P.-P., Chaves L.S.M., Capon A., Pencheon D. (2020). The environmental footprint of health care: A global assessment. Lancet Planet. Health.

[5] Eckelman M.J., Sherman J. (2016). Environmental Impacts of the U.S. Health Care System and Effects on Public Health. PLoS ONE.

[6] Bawaneh K., Nezami F.G., Rasheduzzaman M., Deken B. (2019). Energy Consumption Analysis and Characterization of Healthcare Facilities in the United States. Energies.

[7] MacNeill A.J., Lillywhite R., Brown C.J. (2017). The impact of surgery on global climate: A carbon footprint assessment of operating theatres in three health systems. Lancet Planet. Health.

[8] Smith J.T., Boakye L.A., Ferrone M.L., Furie G.L. (2022). Environmental Sustainability in the Orthopaedic Operating Room. J. Am. Acad. Orthop. Surg..

[9] Rizan C., Bhutta M.F., Reed M., Lillywhite R. (2021). The Carbon Footprint of Waste Streams in a UK Hospital. J. Clean. Prod..

[10] Karnon J., Stahl J., Brennan A., Caro J.J., Mar J., Möller J. (2012). Modeling using discrete event simulation: A report of the ISPOR-SMDM Modeling Good Research Practices Task Force-4. Value Health.

[11] Günal M.M., Pidd M. (2010). Discrete event simulation for performance modelling in health care: A review of the literature. J. Simul..

[12] Vázquez-Serrano J.I., Peimbert-García R.E., Cárdenas-Barrón L.E. (2021). Discrete-Event Simulation Modeling in Healthcare: A Comprehensive Review. Int. J. Environ. Res. Public Health.

[13] Mansouri S.A., Aktas E., Besikci U. (2016). Green Scheduling of a two-machine flowshop: Trade-Off between makespan and energy consumption. Eur. J. Oper. Res..

[14] Saleh J., Mitchell A., Kha S., Outterson R., Choi A., Allen L., Chang T., Ladd A., Goodman S., Fox P. (2023). The Environmental impact of orthopaedic surgery. J. Bone Jt. Surg..

[15] Allihaibi W.G., Masoud M., Elhenawy M., Liu S.Q., Burke J., Karim A. (2021). Solving the emergency care patient pathway by a new integrated simulation–optimisation approach. IEEE Access.

[16] Duma D., Aringhieri R. (2023). Real-time resource allocation in the Emergency Department: A case study. Omega.

[17] Ala A. (2024). A simulation-based optimization evaluation of operating room in healthcare under limitation capacity: A multi-objective approach. Spectr. Eng. Manag. Sci..

[18] Roletto A., Zanardo M., Bonfitto G.R., Catania D., Sardanelli F., Zanoni S. (2024). The environmental impact of energy consumption and carbon emissions in radiology departments: A systematic review. Eur. Radiol. Exp..

[19] Kar E. (2025). Hybrid simulation in healthcare: A systematic exploration of models, applications and emerging trends. J. Simul..

[20] Sahlaoui F.Z., Aboueljinane L., Lebbar M. (2023). A review on simulation-based metamodeling in emergency healthcare: Methodology, applications, and future challenges. Simulation.

[21] Ordu M., Demir E., Tofallis C., Gunal M. (2020). A novel healthcare resource allocation decision support tool: A forecasting-simulation-optimization approach. J. Oper. Res. Soc..

[22] Fanti M.P., Mangini A.M., Dotoli M., Ukovich W. (2013). A three-level strategy for the design and performance evaluation of hospital departments. IEEE Trans. Syst. Man Cybern. Syst..

[23] Traoré M.K., Zacharewicz G., Duboz R., Zeigler B. (2018). Modeling and simulation framework for value-based healthcare systems. Simul. Trans. Soc. Model. Simul. Int..

[24] Rajagopal S.M., Supriya M., Buyya R. (2023). Resource provisioning using meta-heuristic methods for IoT microservices with mobility management. IEEE Access.

[25] Sahoo S., Sahoo B., Turuk A.K. (2021). A learning automata-based scheduling for deadline sensitive task in the cloud. IEEE Trans. Serv. Comput..

[26] Yu K., Chakraborty C., Xu D., Zhang T., Zhu H., Alfarraj O. (2024). Hybrid quantum–classical optimization for low-carbon sustainable edge architecture in RIS-assisted AIoT healthcare systems. IEEE Internet Things J..

[27] Hossam H.S., Abdel-Galil H., Belal M. (2024). An energy-aware module placement strategy in fog-based healthcare monitoring systems. Clust. Comput..

[28] Alimorad A., Maadani M., Mahdavi M. (2021). REO: A reliable and energy efficient optimization algorithm for beacon-enabled IEEE 802.15.4-based WBANs. IEEE Sens. J..

[29] Alatoun K., Matrouk K., Mohammed M.A., Nedoma J., Martinek R., Zmij P. (2022). A novel low-latency and energy-efficient task scheduling framework for Internet of Medical Things in an edge fog cloud system. Sensors.

[30] Arivazhagan N., Somasundaram K., Babu D.V., Nayagam M.G., Bommi R.M., Mohammad G.B., Kumar P.R., Natarajan Y., Arulkarthick V.J., Shanmuganathan V.K. (2022). Cloud-Internet of Health Things (IOHT) Task Scheduling Using Hybrid Moth Flame Optimization with Deep Neural Network Algorithm for E Healthcare Systems. Sci. Program..

[31] Kahwash F., Hammad A.W.A., Abu-Hamd M., Salman A. (2021). Energy consumption in hospitals: Benchmarking and strategies for reduction. Energy Rep..

[32] Psillaki M., Apostolopoulos N., Makris I., Liargovas P., Apostolopoulos S., Dimitrakopoulos P., Sklias G. (2023). Hospitals’ energy efficiency in the perspective of saving resources and providing quality services through technological options: A systematic literature review. Energies.

[33] Almhafdy A., Korany H.Z., Cao S.-J., Yang B. (2024). Airflow distribution in hospital isolation rooms with different ventilation and exhaust vent configurations. Indoor Built Environ..

[34] Vladu A., Ghitea T.C., Daina L.G., Țîrț D.P., Daina M.D. (2024). Enhancing operating room efficiency: The impact of computational algorithms on surgical scheduling and team dynamics. Healthcare.

[35] Faezipour M., Ferreira S. (2018). A system dynamics approach for sustainable water management in hospitals. IEEE Syst. J..

[36] Savio A., Roletto A., Marchi B., Zanoni S. (2024). Towards a Greener Radiology: A Comprehensive Life Cycle Assessment Framework for Diagnostic Imaging. Environ. Clim. Technol..

[37] Rovira-Simón J., Sales-i-Coll M., Pozo-Rosich P., Hueto-Madrid J.A., Cánovas Paradell R., Ochoa de Echagüen Aguilar A., Carbonell-Cobo M., de Castro R., Shaw G. (2023). The Green Surgical Block 4.0: Automation of the operating theatre’s climate conditions through a real-time patient-flow solution. Future Healthc. J..

[38] Roletto A., Verga M., Savio A., Viganò G.L., Zanoni S. (2026). Energy performance of MRI systems: On-site validation and comparison with manufacturer declarations. Eur. Radiol. Exp..

[39] Rizan C., Steinbach I., Nicholson R., Lillywhite R., Reed M., Bhutta M.F. (2020). The Carbon Footprint of Surgical Operations: A Systematic Review. Ann. Surg..

[40] GlobalSurg Collaborative, NIHR Global Health Research Unit on Global Surgery (2023). Reducing the environmental impact of surgery on a global scale: Systematic review and co-prioritization with healthcare workers. Br. J. Surg..

